# White-coat hypertension and incident end-stage renal disease in patients with non-dialysis chronic kidney disease: results from the C-STRIDE Study

**DOI:** 10.1186/s12967-020-02413-w

**Published:** 2020-06-15

**Authors:** Qin Wang, Yu Wang, Jinwei Wang, Luxia Zhang, Ming-hui Zhao, the Chinese Cohort Study of Chronic Kidney Disease (C-STRIDE), Ming-Hui Zhao, Luxia Zhang, Xiaoqin Wang, Jun Yuan, Qiaoling Zhou, Qiongjing Yuan, Menghua Chen, Xiaoling Zhou, Shuxia Fu, Shaomei Li, Yan Zha, Rongsai Huang, Zhangsuo Liu, JunJun Zhang, Li Wang, Lei Pu, Jian Liu, Suhua Li, Zuying Xiong, Wei Liang, Jinghong Zhao, Jiao Mu, Xiyan Lian, Yunjuan Liao, Hua Gan, Liping Liao, Rong Wang, Zhimei Lv, Yunhua Liao, Ling Pan, Xiaoping Yang, Zhifeng Lin, Zongwu Tong, Yun Zhu, Qiang He, Fuquan Wu, Rong Li, Kai Rong, Caili Wang, Yanhui Zhang, Yue Wang, Wen Tang, Hua Wu, Ban Zhao, Rongshan Li, Lihua Wang, Detian Li, Feng Du, Yonggui Wu, Wei Zhang, Shan Lin, Pengcheng Xu, Hongli Lin, Zhao Hu, Fei Pei, Haisong Zhang, Yan Gao, Luying Sun, Xia Li, Wenke Wang, Fengling Lv, Deguang Wang, Xuerong Wang, Dongmei Xu, Lijun Tang, Yingchun Ma, Tingting Wang, Ping Fu, Tingli Wang, Changying Xing, Chengning Zhang, Xudong Xu, Haidong He, Xiaohui Liao, Shuqin Xie, Guicai Hu, Lan Huang

**Affiliations:** 1 Renal Division, Department of Medicine, Peking University First Hospital, Institute of Nephrology, Peking University, Key Laboratory of Renal Disease, National Health and Family Planning Commission of the People’s Republic of China, Key Laboratory of Chronic Kidney Disease Prevention and Treatment, Ministry of Education, Beijing, 100034 China; 2 Center for Data Science in Health and Medicine, Peking University, Beijing, China; 3 Peking-Tsinghua Center for Life Sciences, Beijing, China

**Keywords:** Ambulatory blood pressure monitoring, White coat hypertension, Chronic kidney disease, End-stage renal disease

## Abstract

**Background:**

Controversy remains whether white coat hypertension (WCH) is associated with renal prognosis in patients with chronic kidney disease (CKD).

**Methods:**

In the present multicenter, prospective study, we analyzed data of participants with CKD stage 1–4 from the Chinese Cohort Study of Chronic Kidney Disease (C-STRIDE). WCH was defined according to two criteria as follows: A, clinical blood pressure (BP) ≥ 140/90 mm Hg and average 24-h ambulatory BP < 130/80 mm Hg; B, clinical BP ≥ 130/80 mm Hg and daytime ambulatory BP < 130/80 mm Hg. Renal outcome was defined as initiation of renal replacement therapy. The association of WCH with renal events was evaluated by Cox regression model.

**Results:**

A total of 1714 patients with CKD were included in the present analysis. The mean age of the population was 48.9 ± 13.8 years and 56.8% were men. The mean baseline estimated glomerular filtration rate (eGFR) was 52.2 ± 30.1 ml/min/1.73 m^2^ and urinary protein was 1.0 (0.4, 2.4) g/day. The overall prevalence of WCH was 4.7% and 16.6% according to criteria A and B, respectively. Incidence rates of renal events were 49.58 and 26.51 according to criteria A and B, respectively, per 1000 person-years during a median follow-up of 4.8 years. After full adjustment, WCH was associated with an increased risk of renal event (criterion A: hazard ratio 2.36, 95% confidence interval 1.29–4.34; for criterion B: hazard ratio 1.90, 95% confidence interval 1.04–3.49) compared with patients with normal BP.

**Conclusions:**

WCH is associated with a greater risk for renal events in non-dialysis dependent Chinese patients with CKD.

## Background

Chronic kidney disease (CKD) is a leading public health challenges with a high prevalence of hypertension. Elevated blood pressure (BP) is one of the major contributors to progressive loss of renal function and development of cardiovascular disease (CVD) in patients with CKD [1]. Accurate diagnosis and treatment of hypertension are important in managing patients with CKD. Traditionally, diagnosis and management of hypertension were based on clinical BP (CBP) measurement. Since the development of the ambulatory BP (ABP) monitoring method, which can evaluate BP throughout the 24-h cycle in nonmedical settings, an increasing amount of evidence has suggested that ABP is correlated better with long-term prognosis compared with CBP [2, 3].

Four different BP patterns have been identified by a combination of arbitrary CBP and ABP cut-off values as normal BP (NT), white coat hypertension (WCH), masked hypertension (MH), and sustained hypertension (SH). In contrast to the definite prognostic value of MH and SH, the prognostic value of WCH is still controversial in primary hypertension and hypertensive patients with CKD. WCH is defined as the condition in which CBP, but not out-of-office BP, is elevated. In some studies, WCH was associated with a greater prevalence of target organ damage [4–6] and worse prognosis [7] compared with NT in patients with CKD. However, in other studies, no differences in the risk of end stage renal disease (ESRD) and CVD were reported between these two groups [8, 9].

This study aimed to assess whether WCH is associated with the risk of ESRD in individuals with CKD.

## Methods

### Participants

The Chinese Cohort Study of Chronic Kidney Disease (C-STRIDE) is a large, nationwide, multicenter, prospective cohort study of patients with CKD in which BP was evaluated on the basis of office and out-of-office measurements in a subgroup of patients at enrollment. The C-STRIDE included 39 hospitals located in 22 provinces of China. The design and method of C-STRIDE have been described in detail elsewhere [10–12]. The inclusion criteria, exclusion criteria and baseline characteristics of the cohort are listed in Additional file 1: Table S1. Enrollment started in November 2011 and 3700 patients were enrolled by December 2016. A total of 2114 patients had ABP and CBP measurements at enrollment. Among them, 400 patients were excluded from the present analysis because of invalid ABP and CBP measurements. Finally, the data of 1714 patients with CKD were collected and analyzed. Comparison of baseline characteristics of participants who were included and excluded in the current analysis is shown in Additional file 1: Table S2. The study was approved by the Ethics Committee of Peking University First Hospital and the entire protocol was in adherence with the Declaration of Helsinki. All participants signed written informed consent before data collection.

### Blood Pressure measurements

CBP was measured with a mercury sphygmomanometer for three times after the participants had sat quietly for 5 to 10 min. Measurements were performed by an experienced nurse, who was unaware of the results of ABP readings. The mean of three consecutive readings was recorded as CBP for analysis. Twenty-four-hour ABP monitoring was performed via calibrated devices in each clinic center, with BP readings set at 15-minute intervals from 7:00 am to 10:00 pm and 30-min intervals from 10:00 pm to 7 am. Twenty-four-hour BP, daytime BP, and nighttime BP were defined as the mean value of BP readings during a 24-h cycle, daytime, and nighttime, respectively. Valid measurement was regarded as successful documentation of at least 70% of BP readings taken during a 24-h period. CBP and ABP measurements were taken from the non-dominant arm with an appropriate cuff size.

### Definition of blood pressure patterns

We grouped the patients according to two criteria as follows. (1) In criterion A, conventional criteria based on CBP and 24-h ABP were used as follows: NT, with CBP < 140/90 mm Hg and 24-h ABP < 130/80 mm Hg; WCH, with CBP ≥ 140/90 mm Hg and 24-h ABP < 130/80 mm Hg; MH, with CBP < 140/90 mm Hg and 24-h ABP ≥ 130/80 mm Hg; and SH, with CBP ≥ 140/90 mm Hg and 24-h ABP ≥ 130/80 mm Hg [13–16]. (2) In criterion B, criteria based on CBP and daytime ABP according to the 2017 clinical practice guidelines of the American College of Cardiology (ACC) and the American Heart Association (AHA) were used as follows: NT, with CBP < 130/80 mm Hg and daytime BP < 130/80 mm Hg; WCH, with CBP ≥ 130/80 mm Hg and daytime BP < 130/80 mm Hg; MH, with CBP < 130/80 mm Hg and daytime BP ≥ 130/80 mm Hg; and SH, with CBP ≥ 130/80 mm Hg and daytime BP ≥ 130/80 mm Hg [17].

### Definition of renal end-point events

Renal events were defined as initiation of renal replacement therapy, including dialysis and transplantation. The end-point events were assessed every 3 months, either by phone interviews or routine clinical visits, until 31 December, 2017 in the current analysis. Suspected end-point events were ascertained by an independent end-point assessment committee. The follow-up protocol has been described in detail elsewhere [10].

### Definition of covariate

A smoker was defined as a patient who was currently smoking or had ever smoked. Diabetes mellitus was defined as fasting plasma glucose levels ≥ 7.0 mmol/L or a self-reported history of diabetes or current use of anti-diabetes drugs. A history of CVD was defined as past occurrence of myocardial infarction, admittance to a hospital for congestive heart failure, or severe cardiac arrhythmia incidents (resuscitated cardiac arrest, ventricular fibrillation, sustained ventricular tachycardia, paroxysmal ventricular tachycardia, atrial fibrillation or flutter, severe bradycardia, or heart block). Dyslipidemia was defined by the presence of at least one of the following: serum total cholesterol level ≥ 200 mg/dL (5.2 mM/L), triglycerides ≥ 150 mg/dL (1.7 mM/L), low-density lipoprotein cholesterol ≥ 130 mg/dL (3.4 mM/L), and high-density lipoprotein cholesterol < 40 mg/dL (1.0 mM/L), or current use of lipid-lowering drugs. Anemia was defined as hemoglobin levels < 100 g/L. The glomerular filtration rate (GFR) was estimated from serum creatinine measurements and demographic characteristics by the Chronic Kidney Disease Epidemiology Collaboration equation [18]. Patients were divided into four stages according to the level of estimated GFR (eGFR) as follows: CKD stage 1 (≥ 90 ml/min/1.73 m^2^), CKD stage 2 (60–89 ml/min/1.73 m^2^), CKD stage 3 (30–59 ml/min/1.73 m^2^), and CKD stage 4 (15–29 ml/min/1.73 m^2^) [19].

### Statistical analysis

Continuous variables are presented as means ± standard deviations, while non-parametric variables are expressed as the median and interquartile ranges (IQR). Frequency and proportions were used for categorical variables. We used one-way ANOVA or the Kruskal–Wallis test to compare continuous variables and the Chi square test to compare categorical variables. Comparison between two groups was performed using the independent sample *T* test, Mann–Whitney U test, and Chi square test for continuous variables and categorical variables.

The incidence rate of renal events was calculated as the number of events per 1000 patient-years. The cumulative hazard ratio for four BP patterns was calculated by Kaplan–Meier (KM) curves. Log-rank tests were used to compare event rates among groups.

A multivariable Cox proportional hazards regression model was used to investigate the associations between BP patterns and outcomes. Model 1 was adjusted for age (continuous) and sex (male vs. female), body mass index (continuous), smoking (yes vs. no), previous history of CVD (yes vs. no), diabetes (yes vs. no), antihypertension therapy (yes vs. no), albumin (continuous), dyslipidemia (yes vs no), anemia (yes vs no), causes of CKD (glomerulonephritis vs others, diabetic kidney disease vs others), logarithm-transformed urinary protein (continuous), and eGFR (continuous). Further adjustment was performed with clinic systolic BP and 24‐h systolic BP (continuous) in model 2, with clinic systolic BP and daytime systolic BP (continuous) in model 3, and with clinic systolic BP and nighttime systolic BP (continuous) in model 4. Results of all regression models are reported as hazards ratios (HRs) and 95% confidence intervals (CIs). Missing data were filled with means for continuous variables with a normal distribution and with medians for continuous variables with a non-normal distribution, while categorical variables were filled with a separate category. The proportional hazards assumption was assessed by log-minus-log plots. Sensitivity analyses were performed by a competing risk model to decrease the competing risk of death before ESRD. The interactions of diabetes and glomerulonephritis (GN) with WCH on renal outcome were assessed. Additionally, stratified analysis (patients with diabetes vs. patients without diabetes) was performed.

Data were analyzed using SPSS software (version22.0; IBM Corp. Armonk, NY, USA) and SAS System version 9.4 (SAS Institute, Cary, NC, USA). A two-sided *P *< 0.05 was considered statistically significant.

## Results

### Baseline characteristics

**Fig. 1:**
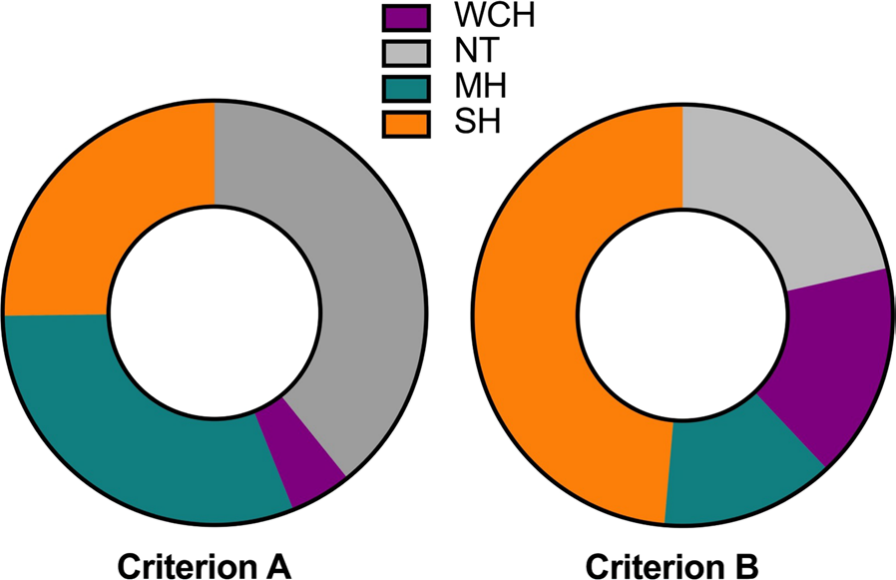
Proportion of BP patterns by different diagnostic criteria

A total of 1714 patients with CKD were included in the present analysis. The mean age of the study population was 48.9 ± 13.8 years and 56.8% were men. Notably, 24.7% of patients had diabetes and 9.0% had a prior history of CVD. A total of 76.7% of patients were taking at least one antihypertensive medication. According to criterion A, there were 672 (39.2%), 81 (4.7%), 529 (30.9%), and 432 (25.2%) patients in the NT, WCH, MH, and SH groups, respectively. The corresponding prevalence changed to 21.4%, 16.6%, 13.4%, and 48.6% according to criterion B in the NT, WCH, MH, and SH groups, respectively (Fig. 1).

The baseline characteristics of the participants according to their BP pattern as defined by criterion A are shown in Table 1. Participants in the WCH, MH, and SH groups were older, had a higher body mass index and urinary protein excretion, had a higher prevalence of diabetes and antihypertensive treatment, and had a lower eGFR compared with those in the NT group (all p < 0.05). Similar characteristics were observed when patients were grouped by criterion B (Additional file 1: Table S3).

**Table 1:** Baseline characteristic of participants according to different BP patterns diagnosed by criterion A

	Total (N = 1714) (N = 1714)	NT (N = 672) (N = 672)	WCH (N = 81) (N = 83)	MH (N = 529) (N = 538)	SH (N = 432) (N = 435)	*P*
Age (years)	48.9 ± 13.8	46.7 ± 14.4	53.1 ± 14.4^a^	49.9 ± 12.8^ab^	50.0 ± 13.3^ab^	< 0.001
Male, n (%)	974 (56.8%)	321 (47.8%)	38 (46.9%)	318 (60.1%)^ab^	297 (68.8%)^abc^	< 0.001
BMI (kg/m^2^)	24.6 ± 3.9	23.9 ± 3.6	25.0 ± 4.0^a^	24.8 ± 3.8^a^	25.6 ± 4.1^ac^	< 0.001
Smokers, n (%)	623 (36.8%)	188 (28.2%)	24 (30.8%)	213 (40.7%)^a^	198 (46.7%)^abc^	< 0.001
DM, n (%)	366 (24.7%)	94 (16.5%)	18 (26.1%)^a^	123 (26.9%)^a^	131 (34.2%)^abc^	< 0.001
CVD history, n (%)	155 (9.0%)	41 (6.1%)	12 (14.8%)	55 (10.4%)	47 (10.9%)	0.004
Anti-hypertension, n (%) treatment	1245 (76.7%)	380 (61.8%)	66 (85.7%)^a^	422 (81.8%)^a^	377 (90.8%)^ac^	< 0.001
Causes of CKD*						< 0.001
DKD	212 (12.4%)	35 (5.2%)	11 (13.6%)^a^	73 (13.8%)^a^	93 (21.5%)^abc^	
GN	1048 (61.1%)	489 (72.8%)	40 (49.4%)^a^	313 (59.2%)^a^	206 (47.7%)^ac^	
Others	442 (25.8%)	144 (21.4%)	29 (35.8%)^a^	140 (26.5%)	129 (29.9%)^a^	
ALB (g/L)	38.3 ± 7.4	38.7 ± 7.2	39.4 ± 5.2	38.2 ± 7.6	37.7 ± 7.9^a^	0.1
FBG (mmol/L)	4.9 (4.4, 5.6)	4.8 (4.3, 5.4)	5.0 (4.5, 5.5)	4.9 (4.4, 5.7)^a^	5.0 (4.5, 5.9)^a^	0.004
HGB (g/L)	126.5 ± 22.4	127.7 ± 19.55	121.9 ± 22.2^a^	126.3 ± 24.2	125.7 ± 24.5	0.1
TG (mmol/L)	1.8 (1.2, 2.5)	1.7 (1.2, 2.4)	1.8 (1.3, 2.4)	1.9 (1.3, 2.6)^a^	1.9 (1.3, 2.6)^a^	0.004
TC (mmol/L)	4.7 (3.9, 5.7)	4.7 (3.9, 5.7)	4.6 (3.8, 5.5)	4.7 (3.9, 5.6)	4.7 (3.9, 5.7)	0.8
HDLC (mmol/L)	1.1 (0.9, 1.3)	1.1 (0.9, 1.4)	1.0 (0.9, 1.3)	1.0 (0.9, 1.3)^a^	1.1 (0.9, 1.2)^a^	0.001
LDLC (mmol/L)	2.6 (2.1, 3.2)	2.6 (2.1, 3.2)	2.6 (2.1, 3.2)	2.5 (2.1, 3.2)	2.7 (2.2, 3.3)	0.5
Cr (μmol/L)	98 (141, 198)	116.0 (80.0, 164.2)	153.0 (106.7, 218.4)^a^	151.0 (108.0, 204.5)^a^	167.0 (122.0, 243.2)^ac^	< 0.001
eGFR (mL/min/1.73 m^2^)	52.2 ± 30.1	62.9 ± 32.7	43.3 ± 24.3^a^	48.2 ± 28.2^a^	42.2 ± 23.3^ac^	< 0.001
24 h-Upro (g/L)	1.0 (0.4, 2.4)	0.7 (0.3, 1.5)	1.0 (0.4, 2.9)^a^	1.1 (0.4, 2.5)^a^	1.8 (0.8, 3.5)^abc^	< 0.001
CKD stages, n (%)						< 0.001
1	256 (14.9%)	174 (25.9%)	5 (6.2%)^a^	55 (10.4%)^a^	22 (5.1%)^ac^	
2	305 (17.8%)	144 (21.4%)	11 (13.6%)	90 (17.0%)	60 (13.9%)^a^	
3	676 (39.5%)	228 (33.9%)	36 (44.4%)	220 (41.6%)^a^	192 (44.5%)^a^	
4	477 (27.8%)	126 (18.8%)	29 (35.8%)^a^	164 (31.0%)^a^	158 (36.6%)^a^	

*BP* blood pressure, *NT* normal BP, *WCH* white-coat hypertension, *MH* masked hypertension, *SH* sustained hypertension, *BMI* Body mass index, *GN* glomerulonephritis, *DKD* diabetic kidney disease, *ALB* serum albumin, *FBG* fasting blood glucose, *DM* diabetes mellitus, *HGB* hemoglobin, *TG* triglyceride, *TC* total cholesterol, *HDLC* high-density lipoprotein cholesterol, *LDLC* low-density lipoprotein cholesterol, *Cr* creatinine, *eGFR* estimated glomerular filtration rate, *24* *h-Upro* 24-hour urinary protein, *CKD* chronic kidney disease, *CVD* cardiovascular disease

Missing counts: BMI 4, ALB 243, smoker 22, CVD history 7, antihypertension treatment: 91, Causes of CKD 12, DM 273, FBG 271, HGB 109, TG 311, TC 311, HDLC 352, LDLC 351, and 24 h-Upro 90

* The diagnosis was made mainly basing on medical history and clinical features, with only 578 patients having renal biopsy confirmation. Among them, 513 patients were diagnosed as GN. IgAN constituted the majority of GN group (54.2%), following by mesangial proliferative glomerulonephritis (32.2%) and membranous nephropathy (10.5%). Others group included hypertensive nephropathy, tubulointerstitial nephritis, and cause unknown etc

^a^*P *< 0.05 comparison with NT

^b^*P *< 0.05 comparison with WCH

^c^*P *< 0.05 comparison with MH

BP parameters are shown in Table 2. According to criterion A, 24-h, daytime, and nighttime systolic BP values were significantly higher in the WCH, MH, and SH groups compared with the NT group (all p < 0.05). According to criterion B, not only systolic BP values, but also 24-h, daytime, and nighttime diastolic BP values were significantly higher in the WCH, MH, and SH groups compared with the NT group (all p < 0.05).

**Table 2:** Clinical and ambulatory BP parameters of patients

	Total (N = 1714)	NT (N = 672)	WCH (N = 83)	MH (N = 538)	SH (N = 435)	*P*
Criterion A						
Clinic SBP (mmHg)	129.5 ± 17.3	118.6 ± 11.1	143.1 ± 13.4^a^	124.9 ± 9.4^ab^	149.4 ± 14.6^abc^	< 0.001
Clinic DBP (mmHg)	80.6 ± 10.4	74.8 ± 7.4	87.9 ± 9.1^a^	78.6 ± 6.5^ab^	90.8 ± 10.5^abc^	< 0.001
24 h-SBP (mmHg)	128.3 ± 17.0	114.5 ± 8.3	118.3 ± 7.3^a^	134.8 ± 13.0^ab^	143.6 ± 15.0^abc^	< 0.001
24 h-DBP (mmHg)	79.3 ± 10.8	70.9 ± 6.0	70.6 ± 5.9	84.5 ± 8.2^ab^	87.4 ± 10.0^abc^	< 0.001
D-SBP (mmHg)	131.1 ± 17.0	116.7 ± 8.9	120.4 ± 7.5^a^	136.4 ± 13.1^ab^	145.1 ± 15.3^abc^	< 0.001
D-DBP (mmHg)	80.7 ± 11.0	72.6 ± 6.3	72.3 ± 6.3	85.8 ± 8.5^ab^	88.6 ± 10.5^abc^	< 0.001
N-SBP (mmHg)	123.6 ± 18.7	109.6 ± 10.0	113.9 ± 10.5^a^	130.4 ± 15.1^ab^	138.9 ± 17.7^abc^	<0.001
N-DBP (mmHg)	75.4 ± 12.5	66.7 ± 7.5	66.6 ± 9.3	80.6 ± 11.1^ab^	84.1 ± 11.1^abc^	< 0.001
Criterion B						
Clinic SBP (mmHg)	129.5 ± 17.3	113.4 ± 9.7	131.6 ± 12.0^a^	117.4 ± 8.9^ab^	139.1 ± 15.9^abc^	< 0.001
Clinic DBP (mmHg)	80.6 ± 10.4	70.7 ± 6.2	83.4 ± 7.0^a^	73.0 ± 5.5^ab^	86.2 ± 9.5^abc^	< 0.001
24 h-SBP (mmHg)	128.3 ± 17.0	112.0 ± 8.3	116.7 ± 8.0^a^	131.5 ± 12.3^ab^	138.6 ± 15.2^abc^	< 0.001
24 h-DBP (mmHg)	79.3 ± 10.8	69.0 ± 5.9	71.7 ± 5.8^a^	81.7 ± 8.1^ab^	85.7 ± 9.5^abc^	< 0.001
D-SBP (mmHg)	131.1 ± 17.0	113.8 ± 8.3	117.7 ± 7.3^a^	133.8 ± 12.2^ab^	140.4 ± 15.1^abc^	< 0.001
D-DBP (mmHg)	80.7 ± 11.0	70.5 ± 5. 9	72.6 ± 5.4^a^	83.7 ± 8.0^ab^	87.1 ± 9.6^abc^	< 0.001
N-SBP (mmHg)	123.6 ± 18.7	107.5 ± 10.8	113.5 ± 10.8^a^	125.4 ± 14.4^ab^	133.6 ± 17.8^abc^	< 0.001
N-DBP (mmHg)	75.4 ± 12.5	64.9 ± 7.5	69.0 ± 7.9^a^	76.9 ± 9.7^ab^	81.9 ± 10.7^abc^	< 0.001

*24* *h-SBP* 24-hour average ambulatory systolic blood pressure, *24* *h-DBP* 24-hour average ambulatory diastolic blood pressure, *D-SBP* daytime systolic blood pressure, *D-DBP* daytime diastolic blood pressure, *N-SBP* nighttime systolic blood pressure, *N-DBP* nighttime diastolic blood pressure

### Survival analysis

The participants were followed up for a total of 7590 years (median: 4.8 years; IQR: 4.0–5.5 years) for renal events. During this period, 286 participants experienced renal event, corresponding to an incidence rate of 37.68 per 1000 person-years. The incidence rate for each BP pattern is shown in Table 3.

**Table 3:** Incidence of renal events by different BP patterns

BP patterns	Number of events	Median follow-up	Events per 1000 person-years	*P* for log-rank
Criterion A				< 0.001
NT (n = 672)	39 (5.80%)	5.1 (4.3, 5.6)	12.08	
WCH (n = 81)	18 (22.22%)	4.9 (3.7, 5.6)	49.58	
MH (n = 529)	111 (20.98%)	4.6 (3.7, 5.5)	49.07	
SH (n = 432)	118 (27.31%)	4.5 (2.7, 5.4)	67.96	
Total	286 (16.69%)	4.8 (4.0, 5.5)	37.68	
Criterion B				< 0.001
NT (n = 367)	17 (4.63%)	5.1 (4.3, 5.6)	9.52	
WCH (n = 284)	35 (12.32%)	5.0 (4.1, 5.5)	26.51	
MH (n = 230)	42 (18.26%)	4.8 (4.0, 5.6)	40.81	
SH (n = 833)	192 (23.05%)	4.5 (3.4, 5.4)	55.57	
Total	286 (16.69%)	4.8 (4.0, 5.5)	37.68	

*BP* blood pressure, *NT* normal BP, *WCH* white-coat hypertension, *MH* masked hypertension, *SH* sustained hypertension

**Fig. 2:**
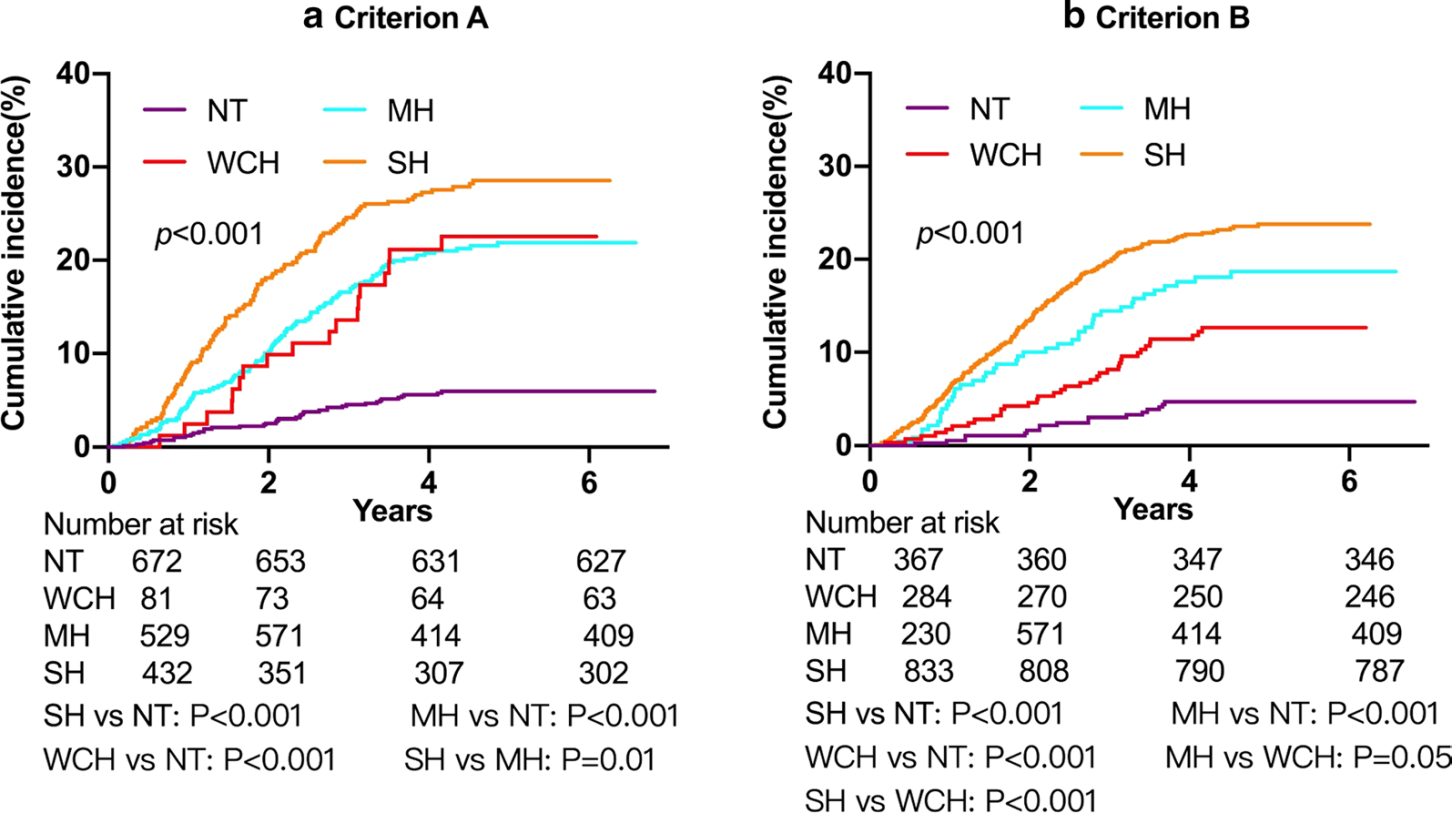
Kaplan–Meier hazard curve for renal events by BP patterns

Patients in the WCH, MH, and SH groups had a higher incidence rates of renal events compared with those in the NT group (Fig. 2). Cox analysis showed that patients in the WCH, MH, and SH groups had a greater risk for renal event, with adjusted HRs of 2.38 (95% CI 1.34–4.23), 2.13 (95% CI 1.45–3.11), and 2.02 (95% CI 1.36–3.00), respectively, as compared with those in NT group by criterion A (Table 4). Similar results were observed by criterion B, with adjusted HRs for the WCH, MH, and SH groups of 1.98 (95% CI 1.10––3.58), 2.24 (95% CI 1.25–3.99), and 2.04 (95% CI 1.21–3.41), respectively (Table 5). The HRs remained significant after further adjustments for clinic systolic BP and 24-h systolic BP, clinic systolic BP and daytime systolic BP, and clinic systolic BP and nighttime systolic BP (Table 4 and Table 5). Sensitivity analyses for decreasing the competing risk of death before ESRD showed consistent results (Additional file 1: Table S4). No interaction between WCH and GN was found (*P* for criterion A: 0.73 and *P* for criterion B: 0.15, respectively). Stratified analysis of the effect of WCH on renal event in patients with diabetes compared with those without diabetes was shown in Additional file 1: Figure S1.

**Table 4:** Hazard ratio for renal events by different BP patterns by criteria A

	Unadjusted	Model 1	Model 2	Model 3	Model 4
	HR (95% CI)	HR (95% CI)	HR (95% CI)	HR (95% CI)	HR (95% CI)
BP patterns					
NT	1.00 (ref)	1.00 (ref)	1.00 (ref)	1.00 (ref)	1.00 (ref)
WCH	4.07 (2.32, 7.11)	2.38 (1.34, 4.23)	2.36 (1.29, 4.34)	2.37 (1.29, 4.35)	2.38 (1.30, 4.37)
MH	3.98 (2.76, 5.73)	2.13 (1.45, 3.11)	2.25 (1.48, 3.41)	2.23 (1.47, 3.37)	2.20 (1.47, 3.30)
SH	5.52 (3.85, 7.93)	2.02 (1.36, 3.00)	2.15 (1.27, 3.63)	2.12 (1.27, 3.57)	2.11 (1.26, 3.54)
Clinic SBP (per 10 mm Hg)	1.28 (1.21, 1.35)	–	1.01 (0.91, 1.11)	1.01 (0.91, 1.11)	1.00 (0.91, 1.11)
24 h-SBP (per 10 mm Hg)	1.32 (1.24, 1.39)	–	0.97 (0.88, 1.07)	–	–
D-SBP (per 10 mm Hg)	1.31 (1.23, 1.38)	–	–	0.98 (0.89, 1.07)	–
N-SBP (per 10 mm Hg)	1.30 (1.23, 1.36)	–	–	–	0.98 (0.90, 1.07)
Age	1.00 (0.99, 1.01)	0.99 (0.97, 0.99)	0.98 (0.97, 0.99)	0.98 (0.97, 0.99)	0.98 (0.97, 0.99)
Sex (M vs W)	1.32 (1.04, 1.68)	1.74 (1.25, 2.43)	1.74 (1.25, 2.42)	1.74 (1.25, 2.43)	1.74 (1.25, 2.42)
BMI	0.95 (0.92, 0.99)	0.97 (0.94, 1.01)	0.96 (0.94, 1.01)	0.97 (0.94, 1.01)	0.98 (0.94, 1.01)
Smoker	1.26 (0.99, 1.60)	0.87 (0.64, 1.18)	0.87 (0.64, 1.19)	0.87 (0.64, 1.19)	0.87 (0.64, 1.19)
DM	1.79 (1.38, 2.32)	1.00 (0.61, 1.63)	1.02 (0.62, 1.66)	1.01 (0.62, 1.65)	1.01 (0.62, 1.66)
CVD history	1.34 (0.96, 1.87)	1.25 (0.87, 1.79)	1.25 (0.86, 1.80)	1.25 (0.86, 1.80)	1.25 (0.87, 1.80)
Anti-hypertensive treatment	0.34 (0.23, 0.51)	0.66 (0.44, 1.00)	0.67 (0.44, 1.01)	0.67 (0.44, 1.01)	0.66 (0.44, 1.00)
Dyslipidemia	0.92 (0.69, 1.24)	0.75 (0.54, 1.02)	0.75 (0.54, 1.03)	0.75 (0.54, 1.03)	0.75 (0.54, 1.03)
ALB	0.96 (0.95, 0.97)	0.96 (0.94, 0.98)	0.96 (0.94, 0.98)	0.96 (0.94, 0.98)	0.96 (0.94, 0.98)
Anemia	3.70 (2.83, 4.83)	1.33 (0.98, 1.80)	1.33 (0.98, 1.82)	1.33 (0.98, 1.82)	1.33 (0.98, 1.82)
lgUpro	4.51 (3.51, 5.81)	2.23 (1.62, 3.08)	2.25 (1.63, 3.10)	2.25 (1.63, 3.10)	2.25 (1.63, 3.10)
eGFR	0.94 (0.93, 0.95)	0.94 (0.93, 0.95)	0.94 (0.93, 0.95)	0.94 (0.93, 0.95)	0.94 (0.93, 0.95)
Causes of CKD					
DKD vs others	2.77 (1.99, 3.88)	1.73 (0.98, 3.04)	1.73 (0.98, 3.04)	1.73 (0.98, 3.04)	1.74 (0.99, 3.07)
GN vs others	0.95 (0.71, 1.27)	1.49 (1.10, 2.04)	1.48 (1.08, 2.02)	1.48 (1.08, 2.03)	1.48 (1.08, 2.03)

*NT* normotension, *WCH* white-coat hypertension, MH masked hypertension, *SH* sustained hypertension, *HR* hazard ratio, *CI* confidence interval, *BMI* body-mass index, *DM* diabetes mellitus, *CVD history* history of CV disease, *ALB* serum albumin, *eGFR* estimated glomerular filtration rate, *CKD* chronic kidney disease, *GN* glomerulonephritis, *DKD* diabetic kidney disease

Model 1: adjusted for age, gender, smoker, BMI, DM, CVD history, anti-hypertensive treatment, Dyslipidemia, ALB, Anemia, logarithm transformed 24 h-urine protein, eGFR and causes of CKD

Model 2: model 1 + clinic systolic blood pressures and 24-h ambulatory systolic blood pressure

Model 3: model 1 + clinic systolic blood pressures and daytime systolic blood pressure

Model 4: model 1 + clinic systolic blood pressures and nighttime systolic blood pressure

**Table 5:** Hazard ratio for renal events by different BP patterns by criteria B

	Unadjusted	Model 1	Model 2	Model 3	Model 4
	HR (95% CI)	HR (95% CI)	HR (95% CI)	HR (95% CI)	HR (95% CI)
BP patterns					
NT	1.00 (ref)	1.00 (ref)	1.00 (ref)	1.00 (ref)	1.00 (ref)
WCH	2.76 (1.55, 4.92)	1.98 (1.10, 3.58)	1.90 (1.04, 3.49)	1.90 (1.04, 3.50)	1.90 (1.04, 3.49)
MH	4.29 (2.44, 7.53)	2.24 (1.25, 3.99)	2.23 (1.22, 4.08)	2.22 (1.21, 4.08)	2.21 (1.22, 3.99)
SH	5.69 (3.46, 9.34)	2.04 (1.21, 3.41)	1.91 (1.08, 3.37)	1.91 (1.08, 3.37)	1.90 (1.08, 3.33)
Clinic SBP (per 10 mm Hg)	1.28 (1.21, 1.35)	–	1.03 (0.94, 1.12)	1.02 (0.94, 1.12)	1.02 (0.94, 1.11)
24 h-SBP (per 10 mm Hg)	1.32 (1.24, 1.39)	–	1.00 (0.91, 1.10)	–	–
D-SBP (per 10 mm Hg)	1.31 (1.23, 1.38)	–	–	1.00 (0.92, 1.10)	–
N-SBP (per 10 mm Hg)	1.30 (1.23, 1.36)	–	–	–	1.01 (0.93, 1.10)
Age	1.00 (0.99, 1.01)	0.98 (0.97, 0.99)	0.98 (0.97, 0.99)	0.98 (0.97, 0.99)	0.98 (0.97, 0.99)
Sex (M vs W)	1.32 (1.04, 1.68)	1.79 (1.29, 2.50)	1.78 (1.28, 2.48)	1.78 (1.28, 2.48)	1.78 (1.28, 2.48)
BMI	0.95 (0.92, 0.99)	0.98 (0.95, 1.01)	0.98 (0.94, 1.01)	0.98 (0.94, 1.01)	0.98 (0.94, 1.01)
Smoker	1.26 (0.99, 1.60)	0.88 (0.64, 1.19)	0.88 (0.64, 1.20)	0.88 (0.64, 1.20)	0.88 (0.64, 1.20)
DM	1.79 (1.38, 2.32)	1.00 (0.61, 1.62)	1.00 (0.61, 1.63)	1.00 (0.61, 1.63)	0.99 (0.61, 1.63)
CVD history	1.34 (0.96, 1.87)	1.27 (0.88, 1.83)	1.28 (0.89, 1.84)	1.28 (0.89, 1.84)	1.28 (0.88, 1.84)
Anti-hypertensive treatment	0.34 (0.23, 0.51)	0.63 (0.42, 0.96)	0.64 (0.42, 0.97)	0.64 (0.42, 0.97)	0.64 (0.42, 0.97)
Dyslipidemia	0.92 (0.69, 1.24)	0.73 (0.53, 1.00)	0.73 (0.53, 1.00)	0.73 (0.53, 1.00)	0.73 (0.53, 1.00)
ALB	0.96 (0.95, 0.97)	0.96 (0.94, 0.98)	0.96 (0.94, 0.98)	0.96 (0.94, 0.98)	0.96 (0.94, 0.98)
Anemia	3.70 (2.83, 4.83)	1.36 (1.00, 1.84)	1.34 (0.99, 1.82)	1.34 (0.99, 1.82)	1.34 (0.99, 1.82)
lgUpro	4.51 (3.51, 5.81)	2.33 (1.69, 3.22)	2.31 (1.67, 3.19)	2.31 (1.67, 3.19)	2.30 (1.66, 3.19)
eGFR	0.94 (0.93, 0.95)	0.94 (0.93, 0.95)	0.94 (0.93, 0.95)	0.94 (0.93, 0.95)	0.94 (0.93, 0.95)
Causes of CKD					
DKD vs others	2.77 (1.99, 3.88)	1.73 (0.98, 3.05)	1.73 (0.98, 3.05)	1.73 (0.98, 3.05)	1.73 (0.98, 3.05)
GN vs others	0.95 (0.71, 1.27)	1.44 (1.06, 1.96)	1.48 (1.08, 2.03)	1.48 (1.08, 2.03)	1.48 (1.08, 2.03)

*NT* normotension, *WCH* white-coat hypertension, *MH* masked hypertension, *SH* sustained hypertension, *HR* hazard ratio, *CI* confidence interval, *BMI* body-mass index, *DM* diabetes mellitus, *CVD history* history of CV disease, *ALB* serum albumin, *eGFR* estimated glomerular filtration rate, *CKD* chronic kidney disease, *GN* glomerulonephritis, *DKD* diabetic kidney disease

Model 1: adjusted for age, gender, smoker, BMI, DM, CVD history, anti-hypertensive treatment, Dyslipidemia, ALB, Anemia, logarithm transformed 24 h-urine protein, eGFR and causes of CKD

Model 2: model 1 + clinic systolic blood pressures and 24-h ambulatory systolic blood pressure

Model 3: model 1 + clinic systolic blood pressures and daytime systolic blood pressure

Model 4: model 1 + clinic systolic blood pressures and nighttime systolic blood pressure

## Discussion

In the present prospective cohort study, patients with CKD stages 1–4 were enrolled and followed for a median duration of 4.8 years to investigate the role of the BP pattern for renal prognosis in patients with CKD. We found that WCH, in addition to MH and SH, were associated with an increased risk for renal events in non-dialysis dependent Chinese patients with CKD. This finding suggests that WCH should not be regarded as irrelevant in clinical practice.

With the introduction of ABP monitoring to clinical practice, four BP patterns have been defined according to the combination of both CBP and ABP readings. The prevalence of WCH in patients with CKD varied in different studies, ranging from 2.3% in the African American Study of Kidney Disease and Hypertension (AASK) study [20], 11% in the German Chronic Kidney Disease (GCKD) study [6], and 31.7% in a Italian study [21]. This difference between studies could be partly ascribed to different classification criteria adopted in different studies, in addition to some specific features, such as race, genetics, etiology of CKD, and comorbidities, of each cohort. The present study showed that the prevalence of WCH was 4.7% when diagnosed by criterion A. This finding is similar to that of 4.1% in the Chronic Renal Insufficiency Cohort (CRIC) Study [4] and 5.6% in the Chronic Kidney Disease Japan Cohort (CKD-JAC) [22], which used the same diagnostic criteria as our study. When AHA/ACC criteria were adopted, the prevalence of WCH increased to 16.6% in the present study, accompanied by corresponding changes in prevalence of NT, MH, and SH.

Unlike MH and SH, which have been proven to have adverse effects on prognosis in patients with CKD, the effect of WCH on long-term prognosis of patients with CKD is still controversial [23, 24]. A subgroup analysis of 4346 patients with CKD from the HONEST study showed that patients with WCH had an increased cardiovascular risk [7]. However, a multicenter cohort study from Italy of 489 patients with CKD that followed patients for a median of 9 months and a single center study of 588 patients with CKD that followed patients for a median of 35 months from Guangzhou, China showed that WCH did not result in adverse prognosis of renal and cardiovascular outcomes [8, 9]. In the present study with more patients enrolled and a longer follow-up period than these previous studies, we found a significant association between WCH and renal events. The risk for renal event in participants with WCH, as defined by either conventional criteria or AHA/ACC criteria, was significantly greater than that of participants with normal BP after full adjustment for relevant confounders. Taken together, these results indicate that WCH may have pathophysiological relevance with the prognosis of CKD.

Patients with WCH had significantly higher clinical and out-of-office BP values compared with normotensive patients in the present study. The relationship between BP and outcomes shows a continuous relationship, even in the normotensive range. Comparatively small increases in mean BP are associated with substantial differences in risk. Cha et al. showed that 24-h ABP progressively increased with the categories of NT to WCH to MH to SH in 1317 patients with CKD [25], which also found in our cohort. A previous study showed an increased cardiovascular risk in patients with WCH when ABP at baseline was markedly higher in the WCH group than in the NT group [26]. Therefore, this difference in absolute BP value between patients with WCH and those with NT might account for, at least in part, the risk of renal events in WCH in the present study. Different diagnostic criteria might affect the difference in BP values between BP patterns. Only a difference in 24-h and/or daytime systolic BP values was found between the WCH and NT groups in the above-mentioned Italian and Guangzhou studies. [8, 9] However, in the present study, 24-h, daytime, and nighttime systolic BP values in patients with WCH, as diagnosed by either criterion A or B, were all significantly higher than those in patients with NT. This more marked difference in BP values between the NT and WCH groups of our cohort might explain the discrepancies between our study and the other studies.

In addition to the different criteria of the definition of WCH, some intrinsic factors of each study (e.g., cause and stage of enrolled participants with CKD, race and ethnicity of the study population, presence of concomitant additional cardiovascular risk factors, treatment status, and follow-up length), might also contribute to the inconsistency in WCH values on prognosis. [27] For instance, the most common cause of CKD in the Italian study was hypertensive nephropathy [8], which accounted for 44.6% of the cohort, and 9.2% had GN. In our study, GN constituted the majority (61.1%) of the cohort, while hypertensive nephropathy, together with other/unknown causes, accounted for 25.8% as shown in Table 1. GN was independently associated with renal progression in the present study, but no interaction between GN and WCH was found. We admitted that the diagnosis of CKD causes was mainly made according to medical history and clinical features with only a few having renal biopsy confirmation. The possibility of some kind of misclassification, therefore, could not be excluded. Whether the cause of CKD plays a role in the association of WCH with progression of CKD remains undefined and requires further study. Additionally, the prevalence of a previous history of CVD and diabetes was 9% and 24.7% in our cohort, respectively, which is markedly lower than that in well-known Western CKD cohorts (30.3% and 36%, respectively in the Italian cohort [8]; 34% and 46%, respectively in CRIC study [4]; and 32% and 35%, respectively in the GCKD study [6]). Since the magnitude of association of CKD with its risk factors is somewhat different between cohorts [28], this difference in prevalence of risk factors between cohorts might also affect the association of WCH with progression of CKD.

This study has some limitations. First, ABP monitoring was only performed once at enrollment. The BP pattern might have changed during follow-up as found in the ELSA study. [29] Furthermore, the APrODiTe-2 study showed that an adverse change (sustained uncontrolled or masked hypertension) of the BP pattern was associated with a change in the eGFR [30]. Therefore, the possibility that patients with WCH and renal events in our cohort might have had their BP pattern changed during follow-up cannot be excluded. Second, not all enrolled patients received ABP monitoring, which is not a mandatory requirement for enrollment of the cohort. This might have resulted in population selection bias. However, patients who were included in the present analysis with ABP data were younger and had high level of eGFR than those who were excluded with a comparable level of urine protein. The presence of these features in patients who were included meant that they had a lower risk of renal progression compared with those who were excluded. Third, although our multivariable analyses included careful adjustment for covariates, we cannot exclude the possibility of residual confounding from other unrecorded covariates that were not ascertained. Forth, doubling of serum creatinine and a rise in proteinuria are also established surrogate renal end-points, which might further refine the effect of hypertension pattern. However, these data were not included in the present analysis. Finally, the cohort comprised only Chinese patients with CKD. As mentioned above, our cohort may have different causes and prevalence of comorbidity of CKD, as well as different ethnic, environmental, and treatment factors, compared with other CKD cohorts from Western countries. Therefore, our results might not be able to be directly extrapolated to other patient populations.

## Conclusions

In conclusion, our study provides evidence that WCH is associated with a greater risk for renal events in non-dialysis dependent Chinese patients with CKD. Future prospective, randomized clinical trials are required to clarify whether treating WCH can delay progression of renal disease in patients with CKD.

## Supplementary information

**Additional file 1: Table S1.** The inclusion and exclusion criteria of C-STRIDE study. **Table S2.** Comparison of baseline characteristics between included and excluded participants in the current study. **Table S3.** Baseline characteristic of participants according to different BP patterns diagnosed by criterion B. **Table S4.** Hazard ratio for renal events by different BP patterns in competing risk model. **Figure S1.** Stratified analysis of the effect of WCH on renal event in patients with diabetes compared with those without diabetes.

## Data Availability

All data generated or analyzed during this study are included in this published article.

## Abbreviations

CKD: Chronic kidney disease
BP: Blood pressure
CBP: Clinical BP
ABP: Ambulatory BP
NT: Normal BP
WCH: White coat hypertension
MH: Masked hypertension
SH: Sustained hypertension
ESRD: End stage of renal disease
CVD: Cardiovascular disease
eGFR: Estimated glomerular filtration rate
C-STRIDE: The Chinese Cohort Study of Chronic Kidney Disease
GCKD: German Chronic Kidney Disease
CRIC: The Chronic Renal Insufficiency Cohort
CKD-JAC: The Chronic Kidney Disease Japan Cohort
ACC: American College of Cardiology
AHA: American Heart Association
IQR: interquartile ranges
KM: Kaplan–Meier
HR: Hazards ratio
CI: Confidence interval
