# Correction to: White-coat hypertension and incident end‑stage renal disease in patients with non‑dialysis chronic kidney disease: results from the C‑STRIDE Study

**DOI:** 10.1186/s12967-020-02447-0

**Published:** 2020-07-13

**Authors:** Qin Wang, Yu Wang, Jinwei Wang, Luxia Zhang, Ming-hui Zhao, the Chinese Cohort Study of Chronic Kidney Disease (C-STRIDE), Ming-Hui Zhao, Luxia Zhang, Xiaoqin Wang, Jun Yuan, Qiaoling Zhou, Qiongjing Yuan, Menghua Chen, Xiaoling Zhou, Shuxia Fu, Shaomei Li, Yan Zha, Rongsai Huang, Zhangsuo Liu, JunJun Zhang, Li Wang, Lei Pu, Jian Liu, Suhua Li, Zuying Xiong, Wei Liang, Jinghong Zhao, Jiao Mu, Xiyan Lian, Yunjuan Liao, Hua Gan, Liping Liao, Rong Wang, Zhimei Lv, Yunhua Liao, Ling Pan, Xiaoping Yang, Zhifeng Lin, Zongwu Tong, Yun Zhu, Qiang He, Fuquan Wu, Rong Li, Kai Rong, Caili Wang, Yanhui Zhang, Yue Wang, Wen Tang, Hua Wu, Ban Zhao, Rongshan Li, Lihua Wang, Detian Li, Feng Du, Yonggui Wu, Wei Zhang, Shan Lin, Pengcheng Xu, Hongli Lin, Zhao Hu, Fei Pei, Haisong Zhang, Yan Gao, Luying Sun, Xia Li, Wenke Wang, Fengling Lv, Deguang Wang, Xuerong Wang, Dongmei Xu, Lijun Tang, Yingchun Ma, Tingting Wang, Ping Fu, Tingli Wang, Changying Xing, Chengning Zhang, Xudong Xu, Haidong He, Xiaohui Liao, Shuqin Xie, Guicai Hu, Lan Huang

**Affiliations:** 1 Renal Division, Department of Medicine, Peking University First Hospital, Institute of Nephrology, Peking University, Key Laboratory of Renal Disease, National Health and Family Planning Commission of the People’s Republic of China, Key Laboratory of Chronic Kidney Disease Prevention and Treatment, Ministry of Education, Beijing, 100034 China; 2 Center for Data Science in Health and Medicine, Peking University, Beijing, China; 3 Peking-Tsinghua Center for Life Sciences, Beijing, China

## Correction to: J Transl Med (2020) 18:238 10.1186/s12967-020-02413-w

After publication of the original article [1], the authors identified errors in Tables 1 and 2. The correct tables are given below.

**Table 1:** Baseline characteristic of participants according to different BP patterns diagnosed by criterion A

	Total (N = 1714)	NT (N = 672)	WCH (N = 81)	MH (N + (N = 529)	SH (N = 432)	*P*
Age (years)	48.9 ± 13.8	46.7 ± 14.4	53.1 ± 14.4^a^	49.9 ± 12.8^ab^	50.0 ± 13.3^ab^	< 0.001
Male, n (%)	974 (56.8%)	321 (47.8%)	38 (46.9%)	318 (60.1%)^ab^	297 (68.8%)^abc^	< 0.001
BMI (kg/m^2^)	24.6 ± 3.9	23.9 ± 3.6	25.0 ± 4.0^a^	24.8 ± 3.8^a^	25.6 ± 4.1^ac^	< 0.001
Smokers, n (%)	623 (36.8%)	188 (28.2%)	24 (30.8%)	213 (40.7%)^a^	198 (46.7%)^abc^	< 0.001
DM, n (%)	366 (24.7%)	94 (16.5%)	18 (26.1%)^a^	123 (26.9%)^a^	131 (34.2%)^abc^	< 0.001
CVD history, n (%)	155 (9.0%)	41 (6.1%)	12 (14.8%)	55 (10.4%)	47 (10.9%)	0.004
Anti-hypertension, n (%) treatment	1245 (76.7%)	380 (61.8%)	66 (85.7%)^a^	422 (81.8%)^a^	377 (90.8%)^ac^	< 0.001
Causes of CKD*						< 0.001
DKD	212 (12.4%)	35 (5.2%)	11 (13.6%)^a^	73 (13.8%)^a^	93 (21.5%)^abc^	
GN	1048 (61.1%)	489 (72.8%)	40 (49.4%)^a^	313 (59.2%)^a^	206 (47.7%)^ac^	
Others	442 (25.8%)	144 (21.4%)	29 (35.8%)^a^	140 (26.5%)	129 (29.9%)^a^	
ALB (g/L)	38.3 ± 7.4	38.7 ± 7.2	39.4 ± 5.2	38.2 ± 7.6	37.7 ± 7.9^a^	0.1
FBG (mmol/L)	4.9 (4.4, 5.6)	4.8 (4.3, 5.4)	5.0 (4.5, 5.5)	4.9 (4.4, 5.7)^a^	5.0 (4.5, 5.9)^a^	0.004
HGB (g/L)	126.5 ± 22.4	127.7 ± 19.55	121.9 ± 22.2^a^	126.3 ± 24.2	125.7 ± 24.5	0.1
TG (mmol/L)	1.8 (1.2, 2.5)	1.7 (1.2, 2.4)	1.8 (1.3, 2.4)	1.9 (1.3, 2.6)^a^	1.9 (1.3, 2.6)^a^	0.004
TC (mmol/L)	4.7 (3.9, 5.7)	4.7 (3.9, 5.7)	4.6 (3.8, 5.5)	4.7 (3.9, 5.6)	4.7 (3.9, 5.7)	0.8
HDLC (mmol/L)	1.1 (0.9, 1.3)	1.1 (0.9, 1.4)	1.0 (0.9, 1.3)	1.0 (0.9, 1.3)^a^	1.1 (0.9, 1.2)^a^	0.001
LDLC (mmol/L)	2.6 (2.1, 3.2)	2.6 (2.1, 3.2)	2.6 (2.1, 3.2)	2.5 (2.1, 3.2)	2.7 (2.2, 3.3)	0.5
Cr (μmol/L)	98 (141, 198)	116.0 (80.0, 164.2)	153.0 (106.7, 218.4)^a^	151.0 (108.0, 204.5)^a^	167.0 (122.0, 243.2)^ac^	< 0.001
eGFR (mL/min/1.73 m^2^)	52.2 ± 30.1	62.9 ± 32.7	43.3 ± 24.3^a^	48.2 ± 28.2^a^	42.2 ± 23.3^ac^	< 0.001
24 h-Upro (g/L)	1.0 (0.4, 2.4)	0.7 (0.3, 1.5)	1.0 (0.4, 2.9)^a^	1.1 (0.4, 2.5)^a^	1.8 (0.8, 3.5)^abc^	< 0.001
CKD stages, n (%)						< 0.001
1	256 (14.9%)	174 (25.9%)	5 (6.2%)^a^	55 (10.4%)^a^	22 (5.1%)^ac^	
2	305 (17.8%)	144 (21.4%)	11 (13.6%)	90 (17.0%)	60 (13.9%)^a^	
3	676 (39.5%)	228 (33.9%)	36 (44.4%)	220 (41.6%)^a^	192 (44.5%)^a^	
4	477 (27.8%)	126 (18.8%)	29 (35.8%)^a^	164 (31.0%)^a^	158 (36.6%)^a^	

* The diagnosis was made mainly basing on medical history and clinical features, with only 578 patients having renal biopsy confirmation. Among them, 513 patients were diagnosed as GN. IgAN constituted the majority of GN group (54.2%), following by mesangial proliferative glomerulonephritis (32.2%) and membranous nephropathy (10.5%). Others group included hypertensive nephropathy, tubulointerstitial nephritis, and cause unknown etc

^a^*P *< 0.05 comparison with NT

^b^*P *< 0.05 comparison with WCH

^c^*P *< 0.05 comparison with MH

**Table 2:** Clinical and ambulatory BP parameters of patients

	Total	NT	WCH	MH	SH	*P*
Criterion A						
Clinic SBP (mmHg)	129.5 ± 17.3	118.6 ± 11.1	143.1 ± 13.4^a^	124.9 ± 9.4^ab^	149.4 ± 14.6^abc^	< 0.001
Clinic DBP(mmHg)	80.6 ± 10.4	74.8 ± 7.4	87.9 ± 9.1^a^	78.6 ± 6.5^ab^	90.8 ± 10.5^abc^	< 0.001
24 h-SBP (mmHg)	128.3 ± 17.0	114.5 ± 8.3	118.3 ± 7.3^a^	134.8 ± 13.0^ab^	143.6 ± 15.0^abc^	< 0.001
24 h-DBP (mmHg)	79.3 ± 10.8	70.9 ± 6.0	70.6 ± 5.9	84.5 ± 8.2^ab^	87.4 ± 10.0^abc^	< 0.001
D-SBP (mmHg)	131.1 ± 17.0	116.7 ± 8.9	120.4 ± 7.5^a^	136.4 ± 13.1^ab^	145.1 ± 15.3^abc^	< 0.001
D-DBP (mmHg)	80.7 ± 11.0	72.6 ± 6.3	72.3 ± 6.3	85.8 ± 8.5^ab^	88.6 ± 10.5^abc^	< 0.001
N-SBP (mmHg)	123.6 ± 18.7	109.6 ± 10.0	113.9 ± 10.5^a^	130.4 ± 15.1^ab^	138.9 ± 17.7^abc^	< 0.001
N-DBP (mmHg)	75.4 ± 12.5	66.7 ± 7.5	66.6 ± 9.3	80.6 ± 11.1^ab^	84.1 ± 11.1^abc^	< 0.001
Criterion B						
Clinic SBP (mmHg)	129.5 ± 17.3	113.4 ± 9.7	131.6 ± 12.0^a^	117.4 ± 8.9^ab^	139.1 ± 15.9^abc^	< 0.001
Clinic DBP (mmHg)	80.6 ± 10.4	70.7 ± 6.2	83.4 ± 7.0^a^	73.0 ± 5.5^ab^	86.2 ± 9.5^abc^	< 0.001
24 h-SBP (mmHg)	128.3 ± 17.0	112.0 ± 8.3	116.7 ± 8.0^a^	131.5 ± 12.3^ab^	138.6 ± 15.2^abc^	< 0.001
24 h-DBP (mmHg)	79.3 ± 10.8	69.0 ± 5.9	71.7 ± 5.8^a^	81.7 ± 8.1^ab^	85.7 ± 9.5^abc^	< 0.001
D-SBP (mmHg)	131.1 ± 17.0	113.8 ± 8.3	117.7 ± 7.3^a^	133.8 ± 12.2^ab^	140.4 ± 15.1^abc^	< 0.001
D-DBP (mmHg)	80.7 ± 11.0	70.5 ± 5. 9	72.6 ± 5.4^a^	83.7 ± 8.0^ab^	87.1 ± 9.6^abc^	< 0.001
N-SBP (mmHg)	123.6 ± 18.7	107.5 ± 10.8	113.5 ± 10.8^a^	125.4 ± 14.4^ab^	133.6 ± 17.8^abc^	< 0.001
N-DBP (mmHg)	75.4 ± 12.5	64.9 ± 7.5	69.0 ± 7.9^a^	76.9 ± 9.7^ab^	81.9 ± 10.7^abc^	< 0.001

*24* *h-SBP* 24-hour average ambulatory systolic blood pressure, *24* *h-DBP* 24-hour average ambulatory diastolic blood pressure, *D-SBP* daytime systolic blood pressure, *D-DBP* daytime diastolic blood pressure, *N-SBP* nighttime systolic blood pressure, *N-DBP* nighttime diastolic blood pressure

The original article has been corrected.
