# Supplementary material for: White-coat hypertension and incident end-stage renal disease in patients with non-dialysis chronic kidney disease: results from the C-STRIDE Study
Source: J Transl Med. 2020 Jun 15;18:238. doi: 10.1186/s12967-020-02413-w (PMC7296682; doi:10.1186/s12967-020-02413-w)

**Additional Material**

Additional file 1: Table S1. The inclusion and exclusion criteria of C-STRIDE study

Additional file 1: Table S2. Comparison of baseline characteristics between included and excluded participants in the current study

Additional file 1: Table S3. Baseline characteristic of participants according to different BP patterns diagnosed by criterion B

Additional file 1: Table S4 Hazard ratio for renal events by different BP patterns in competing risk model

Additional file 1: Figure S1 Stratified analysis of the effect of WCH on renal event in patients with diabetes compared with those without diabetes.

Additional file 1: Table S1. The inclusion and exclusion criteria of C-STRIDE study

| **Criteria** | **Contents** |
| --- | --- |
| **Inclusion criteria** | 1. Aged between 18 and 74 years |
|  | 1. Specified eGFR range according to different CKD etiologies. For patients with glomerulonephritis (GN), the eGFR should be ≥ 15ml·min-1·1.73 m^-2^. For patients with diabetic nephropathy (DN), the defining eligibility was 15ml·min-1·1.73m^-2^ ≤ eGFR < 60ml·min-1·1.73m^-2^ or eGFR ≥ 60ml·min-1·1.73 m^-2^ with “nephrotic range” proteinuria, which is defined as 24-hour urinary protein ≥ 3.5g or urinary albumin creatinine ratio (ACR) ≥ 2000mg/g or corresponding values of urine dipstick test or urinary protein creatinine ratio (PCR). For non-GN and non-DN patients, 15ml·min-1·1.73m^-2^ ≤ eGFR < 60ml·min-1·1.73m^-2^ is setting for enrollment. |
| **Exclusion criteria** | 1. NYHA Class III or IV heart failure |
|  | 1. CKD caused by systemic inflammatory illness or autoimmune disease, such as lupus erythematosus |
|  | 1. Treated with immunosuppressive agents in the preceding 6 months to treat renal or immune disease |
|  | 1. Self-reported or known diagnosis of HIV infection and/or AIDS |
|  | 1. Isolated hematuria |
|  | 1. Self-reported or known diagnosis of cirrhosis |
|  | 1. Pregnant or breast-feeding women |
|  | 1. Malignancy treated with chemotherapy within last 2 years |
|  | 1. Renal or other transplantation |
|  | 1. Hereditary kidney disease |
|  | 1. Participation in intervention clinical trial. |

Additional file 1: Table S2. Comparison of baseline characteristics between included and excluded participants in the current study

|  | Total | Excluded cases | Included cases | *P* |
| --- | --- | --- | --- | --- |
|  | (N=3700) | (N=1986) | (N=1714) |  |
| Age, years | 49.9±14.30 | 50.8±14.7 | 48.9±13.8 | <0.001 |
| Male, n(%) | 2154(58.2%) | 1180(59.4%) | 974(56.8%) | 0.13 |
| Smokers, n(%) | 1230(37.8%) | 607(38.8%) | 623(36.8%) | 0.25 |
| DM, n(%) | 772(24.1%) | 406(23.5%) | 366(24.7%) | 0.43 |
| CVD history, n(%) | 345(9.3%) | 190(9.6%) | 155(9.0%) | 0.59 |
| Causes of CKD |  |  |  | 0.03 |
| DKD | 457(14.0%) | 245(15.6%) | 212(12.5%) |  |
| GN | 1973(60.3%) | 926(58.9%) | 1047(61.6%) |  |
| Others* | 842(25.7%) | 400(25.5%) | 442(26.0%) |  |
| BMI, kg/m^2^ | 24.6±3.6 | 24.4±3.4 | 24.6±3.9 | 0.13 |
| ALB, g/L | 38.7±7.3 | 39.0±7.2 | 38.3±7.4 | 0.001 |
| HGB, g/L | 126.7±22.9 | 127.0±23.2 | 126.5±22.4 | 0.53 |
| TG, mmol/L | 1.8(1.2, 2.5) | 1.8(1.2, 2.6) | 1.8(1.2, 2.5) | 0.77 |
| TC, mmol/L | 4.7(3.9, 5.7) | 4.8(4.0, 5.8) | 4.7(3.9, 5.7) | 0.01 |
| HDLC, mmol/L | 1.1(0.9, 1.3) | 1.1(0.9, 1.3) | 1.1(0.9, 1.3) | 0.39 |
| LDLC, mmol/L | 2.6(2.1, 3.3) | 2.6(2.1, 3.3) | 2.6(2.1, 3.2) | 0.37 |
| eGFR, mL/min/1.73 m^2^ | 49.8±30.3 | 47.8±30.2 | 52.2±30.1 | <0.001 |
| 24h-Upro (g/L) | 1.0(0.4, 2.3) | 1.0(0.4, 2.2) | 1.0(0.4, 2.4) | 0.19 |
| CKD stages |  |  |  | <0.001 |
| 1 | 494(13.4%) | 238(12.0%) | 256(14.9%) |  |
| 2 | 595(16.1%) | 290(14.6%) | 305(17.8%) |  |
| 3 | 1491(40.3%) | 814(41.0%) | 677(39.5%) |  |
| 4 | 1120(30.3%) | 644(32.4%) | 476(27.8%) |  |

Abbreviation: DM: diabetes mellitus, CVD: cardiovascular disease, CKD: chronic kidney disease, DKD: diabetic Kidney Disease, GN: glomerulonephritis, BMI: Body mass index, ALB: serum albumin, HGB: hemoglobin, TG: triglyceride, TC: total cholesterol, HDLC: high‐density lipoprotein cholesterol, LDLC: low‐density lipoprotein cholesterol, 24h-Upro: 24-hour urinary protein, eGFR: estimated glomerular filtration rate,

Missing counts: Smoking 445, BMI 807, ALB 490, HGB 395, TG 732, TC 730, HDLC 835, LDLC 833, DM 493, causes of CKD 428 and 24h-Upro 493

*others including hypertensive nephropathy, tubulointerstitial nephritis, cause unknown etc.

Additional file 1: Table S3. Baseline characteristic of participants according to different BP patterns diagnosed by criterion B

|  | Total(N=1714)  (N=1714) | NT (N=367)  (N=672) | WCH (N=284)  (N=83) | MH (N=230)  (N=538) | SH (N=833)  (N=435) | *P* |
| --- | --- | --- | --- | --- | --- | --- |
| Age (years) | 48.9±13.8 | 45.5±14.1 | 50.6±14.3^a^ | 48.5±13.9^a^ | 49.8±13.2^a^ | <0.001 |
| Male, n(%) | 974(56.8%) | 163(44.4%) | 143(50.4%) | 131(57.0%)^a^ | 537(64.5%)^abc^ | <0.001 |
| BMI (kg/m^2^) | 24.6±3.9 | 23.6±3.5 | 24.8±3.8^a^ | 24.4±3.8^a^ | 25.2±3.9^ac^ | <0.001 |
| Smokers, n(%) | 623(36.8%) | 93(25.4%) | 92(33.0%)^a^ | 80(35.2%)^a^ | 358(43.7%)^abc^ | <0.001 |
| DM, n(%) | 366(24.7%) | 45(14.8%) | 52(21.0%) | 48(23.6%)^a^ | 221(30.6%)^ab^ | <0.001 |
| CVD history, n(%) | 155(9.0%) | 22(6.0%) | 24(8.5%) | 22(9.6%) | 87(10.4%)^a^ | 0.10 |
| Anti-hypertension, n(%) treatment | 1245(76.7%) | 184(55.9%) | 207(76.4%)^a^ | 165(74.3%) | 689(86.0%)^abc^ | <0.001 |
| Causes of CKD |  |  |  |  |  | <0.001 |
| DKD | 212(12.4%) | 18(4.9%) | 24(8.5%) | 30(13.0%)^a^ | 140(16.8%)^ab^ |  |
| GN | 1048(61.1%) | 285(77.7%) | 168(59.2%)^a^ | 148(64.3%)^a^ | 447(53.7%)^ac^ |  |
| Others | 442(25.8%) | 60(16.3%) | 91(32.0%)^a^ | 52(22.6%) | 239(28.7%)^a^ |  |
| ALB (g/L) | 38.3±7.4 | 38.0±7.5 | 39.4±6.4^a^ | 38.9±6.9 | 37.9±7.9^b^ | 0.22 |
| FBG (mmol/L) | 4.9(4.4, 5.6) | 4.8(4.3, 5.4) | 4.9(4.3, 5.4) | 4.9(4.3, 5.5) | 5.0(4.5, 5.9)^a^ | 0.003 |
| HGB (g/L) | 126.5±22.4 | 126.8±18.4 | 127.1±22.3 | 125.7±22.9 | 126.3±24.0 | 0.90 |
| TG (mmol/L) | 1.8(1.2, 2.5) | 1.6(1.1, 2.5) | 1.7(1.2, 2.5) | 1.8(1.14, 2.4) | 1.8(1.3, 2.5)^a^ | 0.23 |
| TC (mmol/L) | 4.7(3.9, 5.7) | 4.6(3.9, 5.6) | 4.7 (3.9, 5.7) | 4.8(4.0, 5.8) | 4.7(3.9, 5.6) | 0.65 |
| HDLC (mmol/L) | 1.1(0.9, 1.3) | 1.1(0.9, 1.4) | 1.1(0.9, 1.3) | 1.1(0.9, 1.3) | 1.0(0.9, 1.2)^a^ | 0.02 |
| LDLC (mmol/L) | 2.6(2.1, 3.2) | 2.6(2.1, 3.2) | 2.7(2.1, 3.2) | 2.6(2.1, 3.1) | 2.6(2.1, 3.2) | 0.86 |
| Cr (μmol/L) | 98(141, 198) | 102(75.5, 146.5) | 138(98, 185)^a^ | 133(101, 189.2) | 159(115, 214.6)^a^ | <0.001 |
| eGFR (mL/min/1.73 m^2^) | 52.2±30.1 | 68.8±33.6 | 51.5±28.3^a^ | 53.0±29.2^a^ | 44.9±26.3^abc^ | <0.001 |
| 24h-Upro (g/L) | 1.0(0.4, 2.4) | 0.8(0.3, 1.8) | 0.7(0.2, 1.7) | 0.9(0.3, 2.1)^a^ | 1.4(0.5, 2.9)^a^ | <0.001 |
| CKD stages, n(%) |  |  |  |  |  | <0.001 |
| 1 | 256(14.9%) | 115(31.3%) | 40(14.1%)^a^ | 29(12.6%)^a^ | 72(8.6%)^ab^ |  |
| 2 | 305(17.8%) | 86(23.4%) | 51(18.0%) | 56(24.3%) | 112(13.4%)^ac^ |  |
| 3 | 676(39.5%) | 110(30.0%) | 114(40.1%)^a^ | 86(37.4%) | 366(43.9%)^ac^ |  |
| 4 | 477(27.8%) | 56(15.3%) | 79(27.8%)^a^ | 59(25.7%)^a^ | 283(34.0%)^ac^ |  |

Abbreviation: BP: blood pressure, NT: normal BP, WCH: white-coat hypertension, MH: masked hypertension, SH: sustained hypertension, BMI: Body mass index, ALB: serum albumin, FBG: fasting blood glucose, DM: diabetes mellitus, HGB: hemoglobin, TG: triglyceride, TC: total cholesterol, HDLC: high‐density lipoprotein cholesterol, LDLC: low‐density lipoprotein cholesterol, Cr: creatinine, eGFR: estimated glomerular filtration rate, 24h-Upro: 24-hour urinary protein, CKD: chronic kidney disease, CVD: cardiovascular disease

Missing counts: BMI 4, ALB 243, smoker 22, CVD history 7, antihypertension treatment: 91, Causes of CKD 12, DM 273, FBG 271, HGB 109, TG 311, TC 311, HDLC 352, LDLC 351, and 24h-Upro 90

^a^ *P*<0.05 comparison with NT

^b^ *P*<0.05 comparison with WCH

^c^ *P*<0.05 comparison with MH

Additional file 1: Table S4 Hazard ratio for renal events by different BP patterns in competing risk model

|  | unadjusted | Model 1 | Model 2 | Model 3 | Model 4 |
| --- | --- | --- | --- | --- | --- |
|  | HR(95%CI) | HR(95%CI) | HR(95%CI) | HR(95%CI) | HR(95%CI) |
| Criteria A |  |  |  |  |  |
| NT (n=475) | 1.00(ref) | 1.00(ref) | 1.00(ref) | 1.00(ref) | 1.00(ref) |
| WCH (n=51) | 4.08(2.36, 7.04) | 2.40(1.41, 4.09) | 2.39(1.35, 4.26) | 2.40(1.35, 4.27) | 2.41(1.36, 4.28) |
| MH (n=335) | 3.95(2.74, 5.68) | 2.11(1.44, 3.09) | 2.25(1.47, 3.42) | 2.22(1.46, 3.38) | 2.19(1.46, 3.30) |
| SH (n=252) | 5.45(3.79, 7.83) | 1.99(1.31, 3.01) | 2.16(1.25, 3.74) | 2.13(1.24, 3.66) | 2.11(1.23, 3.63) |
| Criteria B |  |  |  |  |  |
| NT (n=260) | 1.00(ref) | 1.00(ref) | 1.00(ref) | 1.00(ref) | 1.00(ref) |
| WCH (n=196) | 2.76(1.55, 4.90) | 2.00(1.13, 3.52) | 1.93(1.07, 3.46) | 1.93(1.07, 3.47) | 1.93(1.08, 3.47) |
| MH (n=155) | 4.27(2.44, 7.47) | 2.23(1.26, 3.95) | 2.25(1.23, 4.10) | 2.24(1.23, 4.08) | 2.22(1.24, 3.99) |
| SH (n=502) | 5.64(3.45, 9.24) | 2.01(1.22, 3.33) | 1.92(1.10, 3.35) | 1.91(1.10, 3.34) | 1.90(1.10, 3.29) |

Abbreviation: NT: normotension, WCH: white-coat hypertension, MH: masked hypertension, SH: sustained hypertension, HR: hazard ratio, CI: confidence interval

Model 1: adjusted for age, gender, smoker, BMI, DM, CVD history, anti-hypertensive treatment, Dyslipidemia, ALB, Anemia, logarithm transformed 24h-urine protein, eGFR and causes of CKD

Model 2: model 1 + clinic systolic blood pressures and 24-hour ambulatory systolic blood pressure

Model 3: model 1 + clinic systolic blood pressures and daytime systolic blood pressure

Model 4: model 1 + clinic systolic blood pressures and nighttime systolic blood pressure

Additional file 1: Figure S1 Stratified analysis of the effect of WCH on renal event in patients with diabetes compared with those without diabetes.

Abbreviation: w/o: without, DM: diabetes mellitus


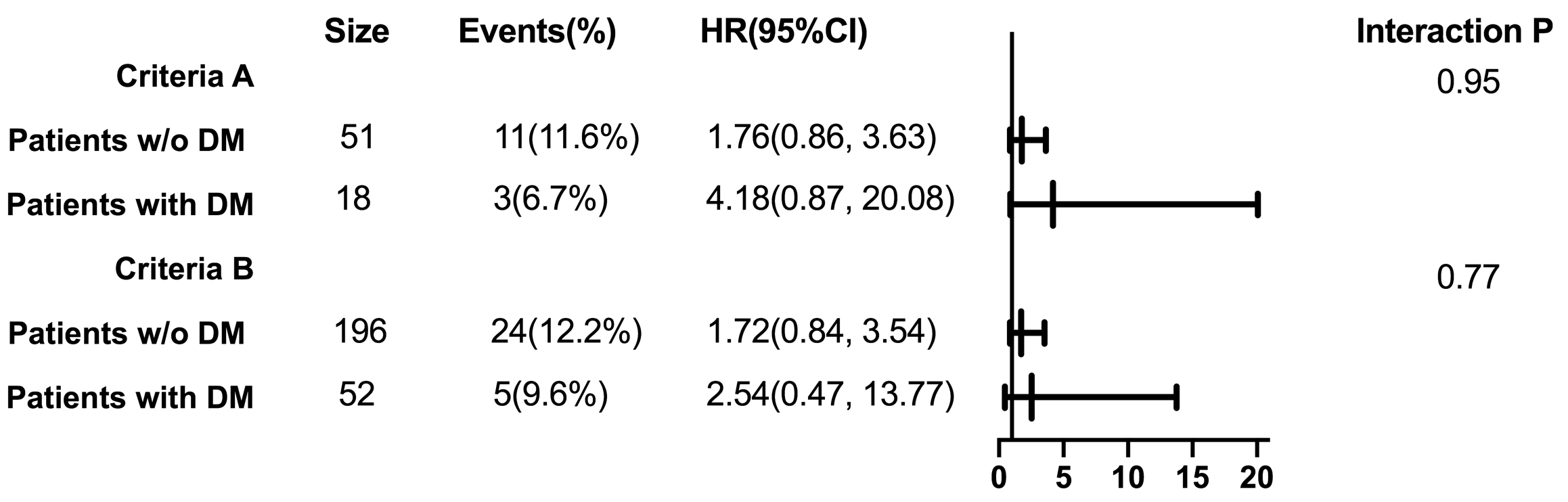

Supplement: Supplementary file 1 — Additional file 1: Table S1. The inclusion and exclusion criteria of C-STRIDE study. Table S2. Comparison of baseline characteristics between included and excluded participants in the current study. Table S3. Baseline characteristic of participants according to different BP patterns diagnosed by criterion B. Table S4. Hazard ratio for renal events by different BP patterns in competing risk model. Figure S1. Stratified analysis of the effect of WCH on renal event in patients with diabetes compared with those without diabetes. [file 12967_2020_2413_MOESM1_ESM.docx]
